# Whole exome sequencing identifies novel germline variants of SLC15A4 gene as potentially cancer predisposing in familial colorectal cancer

**DOI:** 10.1007/s00438-022-01896-0

**Published:** 2022-05-13

**Authors:** Diamanto Skopelitou, Aayushi Srivastava, Beiping Miao, Abhishek Kumar, Dagmara Dymerska, Nagarajan Paramasivam, Matthias Schlesner, Jan Lubinski, Kari Hemminki, Asta Försti, Obul Reddy Bandapalli

**Affiliations:** 1 Molecular Genetic Epidemiology, German Cancer Research Center (DKFZ), Heidelberg, Germany; 2 Medical Faculty Heidelberg, Heidelberg University, Heidelberg, Germany; 3 Institute of Bioinformatics, International Technology Park, Bangalore, India; 4 Manipal Academy of Higher Education (MAHE), Manipal, Karnataka 576104 India; 5 Department of Genetics and Pathology, Pomeranian Medical University in Szczecin, Szczecin, Poland; 6 Computational Oncology, Molecular Diagnostics Program, National Center for Tumor Diseases (NCT), Heidelberg, Germany; 7 Bioinformatics and Omics Data Analytics, German Cancer Research Center (DKFZ), Heidelberg, Germany; 8 Faculty of Medicine and Biomedical Center in Pilsen, Charles University in Prague, 30605 Pilsen, Czech Republic

**Keywords:** SLC15A4, Germline variant, Familial colorectal cancer, Whole exome sequencing

## Abstract

About 15% of colorectal cancer (CRC) patients have first-degree relatives affected by the same malignancy. However, for most families the cause of familial aggregation of CRC is unknown. To identify novel high-to-moderate-penetrance germline variants underlying CRC susceptibility, we performed whole exome sequencing (WES) on four CRC cases and two unaffected members of a Polish family without any mutation in known CRC predisposition genes. After WES, we used our in-house developed Familial Cancer Variant Prioritization Pipeline and identified two novel variants in the solute carrier family 15 member 4 (*SLC15A4*) gene. The heterozygous missense variant, p. Y444C, was predicted to affect the phylogenetically conserved PTR2/POT domain and to have a deleterious effect on the function of the encoded peptide/histidine transporter. The other variant was located in the upstream region of the same gene (GRCh37.p13, 12_129308531_C_T; 43 bp upstream of transcription start site, ENST00000266771.5) and it was annotated to affect the promoter region of *SLC15A4* as well as binding sites of 17 different transcription factors. Our findings of two distinct variants in the same gene may indicate a synergistic up-regulation of *SLC15A4* as the underlying genetic cause and implicate this gene for the first time in genetic inheritance of familial CRC.

**Supplementary Information:**

The online version contains supplementary material available at 10.1007/s00438-022-01896-0.

## Introduction

Several studies have estimated that around 15% of colorectal cancer (CRC) patients show a first-degree family history of colorectal malignancies (Ponz de Leon et al. 1989; Hemminki et al. 2008; Frank et al. 2015; Chau et al. 2016). Analyzing the underlying heritable and environmental factors in twins from Sweden, Denmark, and Finland, Lichtenstein et al. have estimated that genetic factors account for up to 35% of the CRC risk (Lichtenstein et al. 2000). Nevertheless, only a small proportion of familial CRC cases can be traced back to germline mutations in established CRC-predisposing genes. In the present study, we used a family-based whole-exome sequencing approach to fill in this gap and to identify novel CRC predisposition genes with high-to-moderate penetrance germline variants.

The early-identified traditional CRC susceptibility genes include *APC* and mismatch repair genes (*MLH1, MSH2, MSH6, PMS2*), *MUTYH* and *SMAD4/BMPR1A*. Later on, sequencing studies have identified novel predisposition genes for CRC, such as *NTHL1, RNF43, POLE, POLD1, FAN1* and *RPS20* (Jasperson et al. 2010; Briggs and Tomlinson 2013; Palles et al. 2013; Gala et al. 2014; Nieminen et al. 2014; Kuiper and Hoogerbrugge 2015; Segui et al. 2015; Weren et al. 2015; Yan et al. 2017; Lorans et al. 2018; Valle et al. 2019). Further candidate genes recently suggested by modern next generation sequencing methods include the solute carrier (SLC) family of membrane transport genes: *SLC5A9* (p.G492Afs*13), *SLC26A8* (p.R954C) and *SLC11A1* (p.P64A) (Hansen et al. 2017; Yu et al. 2018). Additionally, germline deletions affecting the open reading frame of *SLC18A1* gene have been reported to increase the risk of CRC and lower SLC18A1 protein expression has been further associated with poor clinical outcome (Zhang et al. 2017).

Despite novel findings of predisposition gene candidates in CRC, there still exist 75% of unexplored familial CRC cases. This proportion of familial CRC with unknown genetic background may be accounted for by two major components: either following a monogenic inheritance model based on a single high-penetrance mutation or a polygenic inheritance model based on the combination of multiple low/moderate-penetrance risk alleles (Zetner and Bisgaard 2017). Assuming the monogenic disease model for CRC cases with strong familial clustering, the identification of rare highly penetrant germline variants within pedigree-based studies constitutes a promising approach for elucidating the remaining genetic burden of familial CRC.

For this purpose, we performed whole exome sequencing (WES) on a Polish family with CRC aggregation over three generations. Sequencing data of four CRC cases and two unaffected family members were subsequently analyzed using our in-house developed Familial Cancer Variant Prioritization Pipeline (FCVPPv2) which was used earlier in identification of variants and pathways involved in several familial cancers (Bandapalli et al. 2018; Kumar et al. 2018; Srivastava et al. 2019; Srivastava et al. 2020a; Srivastava et al. 2020b; Skopelitou et al. 2021; Srivastava et al. 2021). Further in silico analyses resulted in the prioritization of a novel missense variant in the solute carrier family 15 member 4 gene (*SLC15A4*), encoding a proton-dependent peptide/histidine transporter. By being involved in multiple signaling pathways regulating cytokine production and thus innate immune responses, SLC15A4 has been shown to promote colitis in an in vivo mouse model (Sasawatari et al. 2011; Kobayashi et al. 2014). Since high expression of the encoded membrane transporter has been further reported in the feces of CRC patients as well as in early-stage CRC cell lines, an important role of SLC15A4 in initial inflammation-induced colorectal carcinogenesis has been suggested (Lee et al. 2016). In this study, we conducted in silico analyses as well as further literature search to link the function of the SLC15A4 protein to a genetic basis, potentially contributing to CRC development in the studied family. By identifying and analyzing an additional variant in the upstream region of the *SLC15A4* gene showing the same familial segregation, we aimed to expand the theory of high-penetrance monogenic inheritance to a synergistic model of coding and non-coding variants underlying cancer predisposition.

## Materials and methods

### Patient samples and ethical permissions

**Fig. 1:**
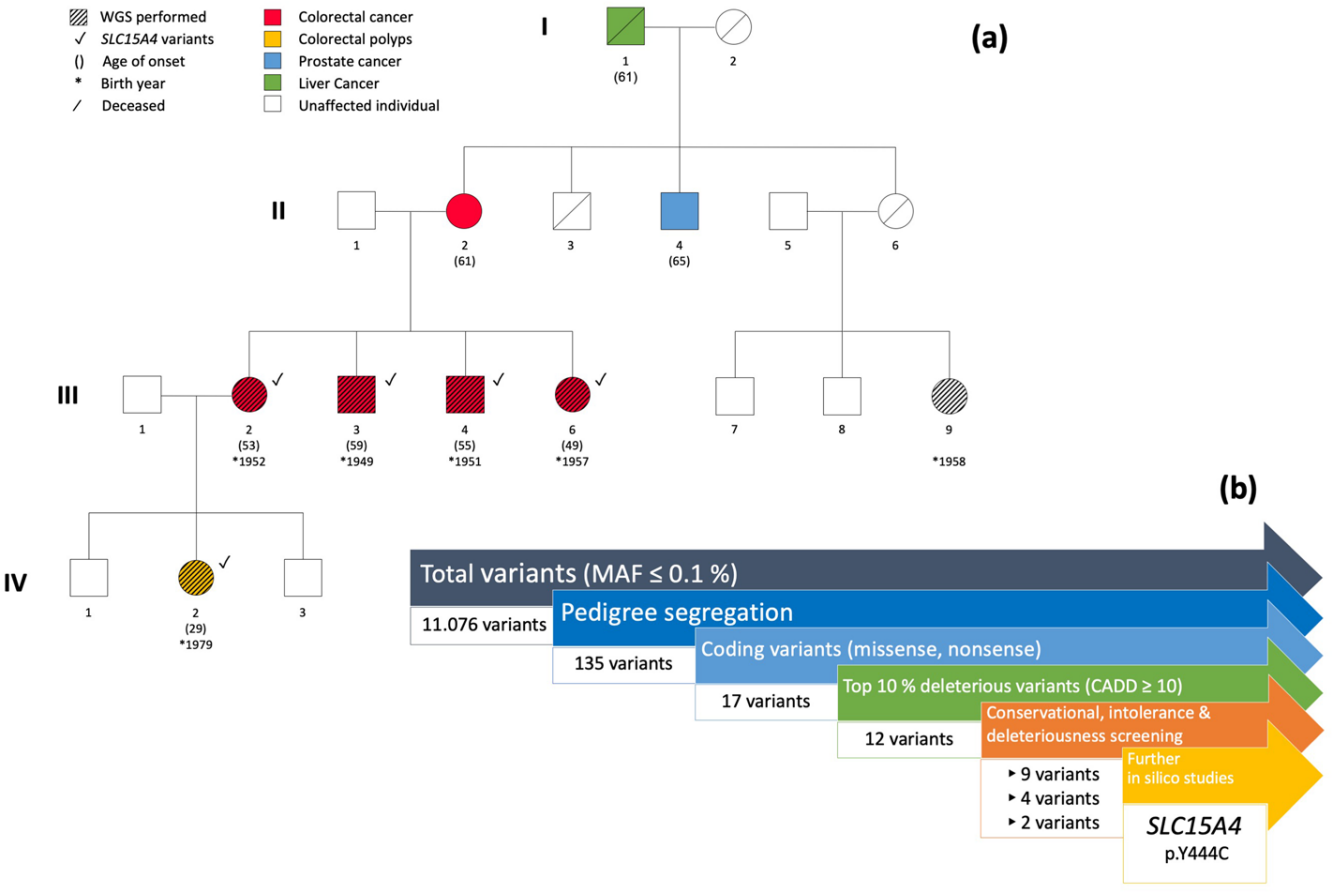
**a** Pedigree of the studied family with CRC aggregation over three generations and the presence of the missense and upstream variants in the *SLC15A4* gene **b** Graphical overview of the filtering process according to the Familial Cancer Variant Prioritization Pipeline version 2 (FCVPPv2)

The family with CRC history over three generations was recruited from Poland (Fig. 1a). Six family members were included in our experiments: four siblings diagnosed with CRC, a child of one CRC case with colorectal polyps and a healthy cousin of the CRC cases. The family was screened for alterations in *APC*, the mismatch repair genes *MLH1, MSH2, MSH3*, large deletions in *EPCAM* and *POLE* p. Leu424Val, *POLD1* p.Ser478Asn and *NTHL1* p.Gln90* mutations and found to be negative. Collection of blood samples and clinical information from subjects was undertaken with informed written consent in agreement with the tenets of the declaration of Helsinki. The study was approved by the Bioethics Committee of the Pomeranian Medical Academy in Szczecin (protocol code No: BN-001/174/05).

### Whole exome sequencing, variant calling and annotation

Genomic DNA was isolated with a modified Lahiri and Schnabel method (Lahiri and Schnabel 1993) and WES was performed using Illumina-based small read sequencing. After mapping to the human reference genome (assembly GRCh37 version Hs37d5) by means of BWA (Li and Durbin 2009), duplicates were removed with Picard (http://broadinstitute.github.io/picard/). SAM tools (Li 2011) and Platypus (Rimmer et al. 2014) were used for calling single nucleotide variants (SNVs) as well as short insertions and deletions (indels), respectively. Variants were then annotated by ANNOVAR (Wang et al. 2010), 1000 Genomes Project (Genomes Project et al. 2015), dbSNP (Smigielski et al. 2000) and Exome Aggregation Consortium (ExAC) (Lek et al. 2016). To be further processed, variants should have a quality score of ≥ 20 and a coverage score of ≥ 5 ×, SNVs should pass the strand bias filter (a minimum one read support from both forward and reverse strand) and indels should pass all the Platypus internal filters. Based on minor allele frequencies (MAFs) deduced from the 1000 Genomes Project Phase 3, non-TCGA ExAC data, NHLBI-ESP6500 and local data sets, rare variants with a MAF ≤ 0.1% in the European population were retained for further analysis. We checked for potential sample swaps and family relatedness by pairwise comparison of the shared rare variants.

### Coding variant analysis according to the FCVPPv2

The resulting variants were analyzed based on our in-house developed FCVPPv2 (Kumar et al. 2018). First, variants were filtered according to the pedigree segregation of the malignancy. Variants should be present in family members affected by CRC and absent in the healthy family member. Since colorectal polyps at a relatively young age may represent a preliminary stage of familial CRC, the respective family member could be a possible carrier and show either presence or absence of the variant of interest.

Of the coding variants fulfilling the pedigree segregation criteria, the most deleterious 10% were retained for further analysis, represented by a PHRED-like CADD score ≥ 10 (Kircher et al. 2014; Rentzsch et al. 2019). To evaluate the evolutionary conservation as an indicator for functional importance of a genomic position, the following scoring tools were applied with respective cutoff values given in brackets: Genomic Evolutionary Rate Profiling (GERP; ≥ 2.0), PhastCons (> 0.3) and PhyloP score (≥ 3.0) (Cooper et al. 2005; Siepel et al. 2005; Pollard et al. 2010). Next, the intolerance of genes against functional genetic variation was assessed by using three intolerance scores (< 0) based on allele frequency data from our in-house datasets, from NHLBI-ESP6500 and ExAC (Petrovski et al. 2013). In the course of intolerance screening, missense and loss-of-function variants were further annotated by the *Z* Score (> 0) and pLI score (≥ 0.9), respectively, which were specifically developed by the ExAC consortium for the particular type of variants (Lek et al. 2016). Last, we evaluated the deleteriousness of non-synonymous and splice site SNVs by applying ten different scoring tools accessed from dbNSFP v3.0 (database for nonsynonymous SNPs’ functional predictions): Sorting Tolerant From Intolerant (SIFT), Polymorphism Phenotyping v2 (PolyPhen-2) HumDiv, PolyPhen-2 HumVar, Log ratio test (LRT), MutationTaster, MutationAssessor, Functional Analysis Through Hidden Markov Models (FATHMM), Reliability Index, Variant Effect Scoring Tool version 3 (VEST3) and Protein Variation Effect Analyzer (PROVEAN) (Liu et al. 2016).

Summarizing, variants with a PHRED-like CADD-score of ≥ 10 as well as ≥ 2 out of the three conservational tools, ≥ 60% of the four intolerance scores and ≥ 60% of the 10 deleteriousness scores fulfilling the selection criteria were retained as the top exonic candidates. Allele frequencies were re-evaluated by means of the gnomAD browser (https://gnomad.broadinstitute.org/) (Karczewski et al. 2019). Since the studied CRC family originates from Poland, the non-Finnish European (NFE) population was taken as the representative population on a large scale.

We further assessed the potential of the variants for being cancer drivers in CRC by checking overall somatic alteration frequencies according to cBioPortal and TCGA PanCancer Atlas, comprising data of 594 CRC patients (Cancer Genome Atlas Research et al. 2013; Gao et al. 2013). Moreover, protein expression levels in CRC tissue were accessed from The Human protein atlas (http://www.proteinatlas.org) (Uhlen et al. 2017).

### Additional in silico analyses based on protein function and phylogenetic conservation

The potential impact of the top missense variants on protein function was assessed by means of Snap^2^ (Hecht et al. 2015). Based on a neural network, Snap^2^ calculates the likelihood of single amino acid substitutions to alter protein function, giving scores between − 100 (low) and + 100 (high). The predicted functional impact is represented in form of heat maps covering each possible amino acid substitution at each position.

Since predictions of the functional impact of variants are based on evolutionary information, we further checked phylogenetic conservation of the top variants among different vertebrate species. Multiple protein sequences of the candidate genes and their orthologs were derived from the National Center for Biotechnology Information (NCBI) (Coordinators 2018) and aligned using COBALT, a constraint-based multiple alignment tool (Papadopoulos and Agarwala 2007). Visualized alignments were manually checked for conservation at the mutation sites and the surrounding regions and percent identity of protein sequences was further calculated by NCBI BLAST (Basic Local Alignment Search Tool). Details of multiple sequence alignment including selected representative species and NCBI accessions of respective genes and their orthologs are summarized in Online Resource 1.

We checked recent literature for established gene-cancer associations, postulated oncogene or tumor-suppressor roles as well as potential cancer-promoting protein functions of the top candidates. Considering the entirety of derived information and in silico analysis results, the candidates showing the most promising impact on protein function or gene regulation were prioritized as the potentially cancer-causing variants in the studied family. Familial segregation of the top-listed variants with the disease was confirmed by visually checking WES data with the help of the Integrative Genomics Viewer (IGV) (Robinson et al. 2017).

### Analysis of regulatory elements and prediction of transcription factor binding sites in the non-coding regions

To assess the biological function and to identify potentially active regulatory regions, the chromatin state of specific genomic positions was predicted by the updated version of CADD (v1.6). For this purpose, CADD v1.6 provides chromHmm and Segway data, which annotate the chromatin state based on large-scale functional genomics datasets such as ChIP-seq data (Ernst and Kellis 2012; Hoffman et al. 2012; Roadmap Epigenomics et al. 2015). Using the intersect function of the Bedtools as well as FANTOM5 and SEA databases, we further scanned for potentially affected regulatory elements such as promoters, enhancers and super-enhancers (Lizio et al. 2015; Wei et al. 2016). Moreover, transcription factor binding sites (TFBSs) were predicted by means of Jaspar2020 with the default relative profile score threshold of 80% and compared between wild-type and mutant sequence (Fornes et al. 2020). Details on the regulatory annotations are provided in a systematic review of in silico prioritization of non-coding variants (Lee et al. 2018).

## Results

### Application of the FCVPPv2 results in the prioritization of two coding variants in PTGES and SLC15A4 genes

The studied family was diagnosed with CRC over three generations, as represented in the pedigree (Fig. 1a). Four siblings affected by CRC in the second generation at the age of < 60 years were considered as cases and should therefore carry the variant of interest. Similarly, the daughter (IV2) of one of the cases (III2) developed colorectal polyps at the relatively young age of 29 years, potentially representing a preliminary stage of familial CRC. Considering the option of having inherited the variant of interest, IV2 was defined as a possible carrier and may present the variant as well. In contrast, an unaffected first cousin of the four CRC cases of a similar age and with healthy parents served as a control and should thus not carry the variant.

Analysis of WES data was performed using our in-house developed FCVPPv2, as visually summarized in Fig. 1b. Of the totally identified 11,076 variants with a MAF ≤ 0.1%, only 135 variants fulfilled the pedigree segregation criteria. Exclusion of intergenic and intronic variants resulted in 28 variants in the coding region and 43 variants located in the non-coding region near transcription start and end sites (5′ and 3′ untranslated regions, upstream and downstream regions). Due to their less pathogenic character, synonymous variants were excluded, leaving 17 missense or nonsense variants for further analysis. 12 of the remaining coding variants reached a PHRED-like CADD score ≥ 10, representing the most deleterious 10% of the variants in the human genome. Application of conservational, intolerance and deleteriousness scores further narrowed down the number of variants to 9, 4 and 2, respectively. The two final missense variants were located in solute carrier family 15 member 4 gene (*SLC15A4*, p.Y444C) and prostaglandin E synthase gene (*PTGES*, p.A133T) and are summarized with respective analysis results in Table 1.

**Table 1:** Overview of the top exonic variants prioritized in the studied CRC family

Gene name	Chromosomal position	Exonic classification	Pedigree segregation	NFE allele frequency		CADD SCORE	Conservational scores			Intolerance scores (%)	Deleteriousness scores^a^ (%)	Amino acid change	Snap^2^		Protein function
				ExAC	gnomAD		GERP + +	PhyloP	PhastCons				Effect score	Accuracy (%)	
PTGES	9_132501952_C_T	Nonsyn SNV	III2, III3, III4, III5, IV2	2.10 × 10^–4^	8.43 × 10^–5^	34	4.67	7.723	1	75	80	A133T	16	59	Glutathione-dependent prostaglandin E synthase, involved in inflammatory responses, fever, pain
SLC15A4	12_129285482_T_C	Nonsyn SNV	III2, III3, III4, III5, IV2	0	0	23.7	5.49	5.609	1	100	90	Y444C	44	71	Proton-dependent peptide/histidine transporter, regulation of innate immune responses

Chromosomal position, classification, pedigree segregation, allele frequency in the Non-Finnish European (NFE) population, PHRED-like CADD score, conservational score and the percentage of reached intolerance and deleteriousness scores are summarized for each variant. Snap^2^ results for the predicted amino acid changes are included with calculated effect scores and accuracies given in %. Respective protein functions of the encoded gene products are derived from Genecards (Stelzer et al. 2016). Non-syn SNV-non-synonymous single nucleotide variant

^a^Following predictions given by deleteriousness scores were considered as favorable in our analysis: SIFT–Damaging (D); Polyphen2_HumDiv, Polyphen2_HumVar–Probably damaging (D) and Possibly damaging (P); LRT–Deleterious (D); MutationTaster–Disease causing (D) and disease causing automatic (A); MutationAssesor–High (H) and medium (M); FATHMM–Damaging (D); MetaSVM–Damaging (D); MetaLR–Damaging (D); Reliability Index ≥ 5; VEST3 ≥ 0.5; PROVEAN–Damaging (D)

*PTGES* encodes a glutathione-dependent synthase catalyzing the oxidoreduction to prostaglandin E2. By playing a role in inflammatory responses, fever and pain, PTGES protein has been reported to be involved in inflammatory diseases such as collagen-induced arthritis and gastritis (Gudis et al. 2005; Korotkova et al. 2011). Similarly, the gene product encoded by *SLC15A4* regulates innate immune responses. Being a proton-dependent peptide/histidine transporter, SLC15A4 protein controls the transport of various molecules from the inside of endosomes to the cytosol and has been associated inter alia with systemic lupus erythematosus (Wang et al. 2013; Lee et al. 2014; Zuo et al. 2014; Zhang et al. 2016).

According to the gnomAD browser, both top-listed variants showed very low allele frequencies in the general NFE population: the *PTGES* variant was annotated with a frequency of around 8.4 × 10^–5^ and the *SLC15A4* variant even less with 0 counts in 113,688 alleles. Moreover, only one allele of the *SLC15A4* variant has been reported in the worldwide population accessed by gnomAD browser, counting in total 251,362 alleles (Karczewski et al. 2019). For further validation of allele frequencies, population data of Trans-Omics for Precision Medicine (TOPMed), integrating large-scale whole genome sequencing data, was checked and did not report the identified *SLC15A4* variant, confirming again its low allele frequency (Li et al. 2020; Taliun et al. 2021).

### Higher alteration frequency and protein expression of SLC15A4 in CRC compared to PTGES

**Fig. 2:**
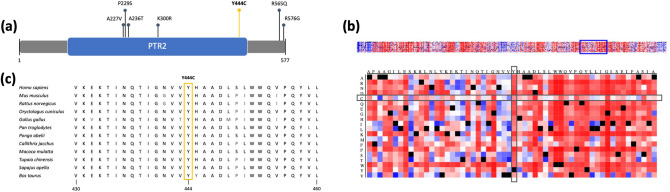
In silico analysis results of the *SLC15A4* variant p.Y444C **a** Graphical overview of the SLC15A4 protein with the PTR2 domain. Somatic mutations identified in CRC were extracted from cBioPortal (www.cbioportal.org) on 13th of December 2020 using the TCGA PanCancer data and are represented by dark pins. The germline missense variant identified in the studied CRC family is highlighted in the form of a yellow pin. **b** Snap^2^ heatmap depicting the functional impact of amino acid substitutions. The missense mutation p.Y444C is highlighted by grey boxes. **c** Extract of multiple sequence alignment of amino acids 430–460 of *SLC15A4* and orthologs. The mutation site is highlighted by a yellow box

**Fig. 3:**
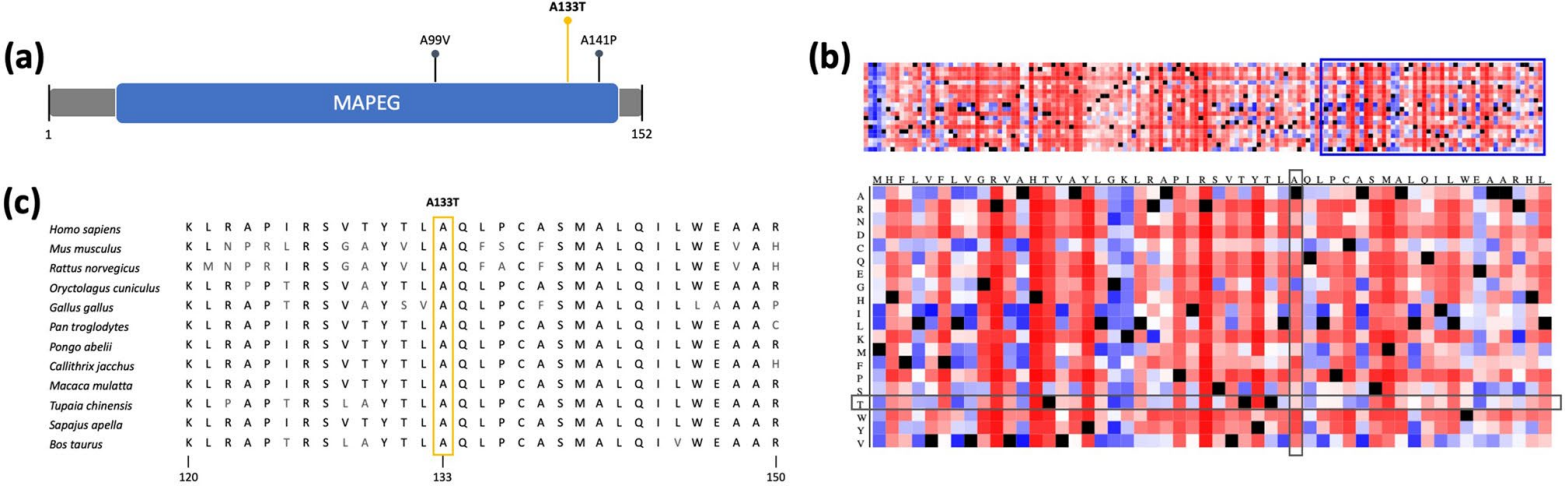
In silico analysis results of the *PTGES* variant p.A133T **a** Graphical overview of the PTGES protein with MAPEG domain. Somatic mutations identified in CRC are extracted from cBioPortal (www.cbioportal.org) on 13th of December 2020 using the TCGA PanCancer data and are represented by dark pins. The germline missense variant identified in the studied CRC family is highlighted in the form a yellow pin. **b** Snap^2^ heatmap depicting the functional impact of amino acid substitutions. The missense mutation p.A133T is highlighted by grey boxes. **c** Extract of multiple sequence alignment of amino acids 120–150 of *PTGES* and orthologs. The mutation site is highlighted by a yellow box

We next checked available CRC patient data for overall somatic gene alteration frequencies to assess the potential of the top candidates for being cancer drivers in CRC. cBioPortal recorded six somatic missense mutations in the *SLC15A4* gene (frequency = 1.01%, Fig. 2a) and only two somatic mutations in the *PTGES* gene (frequency = 0.34%, Fig. 3a) identified within 594 colorectal adenocarcinoma samples from the TCGA PanCancer Atlas. Regarding the overall somatic alteration frequency in all listed cancers, *SLC15A4* showed a generally higher frequency with up to 5.48% in uterine cancer (Online Resource 2a), whereas the maximum alteration frequency of the *PTGES* gene was only 1.7%, also in uterine cancer (Online Resource 2b) (Cancer Genome Atlas Research et al. 2013; Gao et al. 2013). Besides genetic alterations documented in CRC, we checked protein expression levels in CRC samples. According to the Human Protein Atlas, 4 out of 12 investigated CRC samples showed a medium expression of the SLC15A4 protein, whereas 0 out of 11 CRC samples showed a high or medium expression of the PTGES protein (Uhlen et al. 2017).

### In silico analyses predict functional consequences of the SLC15A4 variant on protein level

The identified *SLC15A4* variant p.Y444C was predicted to affect the PTR2 (peptide transport) domain (p.104–495) of the POT (proton-dependent oligopeptide transporter) family (Fig. 2a) and in particular a non-cytoplasmic loop of the SLC15A4 transporter protein, which comprises in total 12 transmembrane domains according to Interpro (Blum et al. 2020). Analysis of the potential impact of the *SLC15A4* missense variant on protein function by means of Snap^2^ resulted in a predicted effect score of 44 with an accuracy of 71% (Fig. 2b). In contrast, the missense variant p.A133T in the *PTGES* gene, affecting the cytoplasmic part of the MAPEG (membrane-associated proteins in eicosanoid and glutathione metabolism) domain (p.17–146) of the PTGES protein (Fig. 3a), was annotated by Snap^2^ with a score of 16 and an accuracy of 59% (Fig. 3b) (Hecht et al. 2015). Due to a higher effect score and accuracy of the prediction in cross-validation, the functional impact of the *SLC15A4* variant was expected to be of higher relevance.

Sequence alignment of the orthologs showed for both variants a universally conserved position with an overall high conservation of the surrounding region among the selected vertebrate species (Figs. 2c, 3c, respectively). Focusing on the five directly adjacent upstream and downstream amino acid positions, multiple sequence alignment resulted in 95.86% identity of the *SLC15A4* and 82.64% identity of the *PTGES* gene with their orthologs. Based on this observation, a higher phylogenetic conservation in the region surrounding the mutation site can be assumed for *SLC15A4*.

The entirety of the in silico analyses led to the prioritization of the missense variant in *SLC15A4* gene (p.Y444C). Familial segregation of this variant was manually checked and confirmed by applying IGV on the WES data.

### Identification of an additional variant at an active transcription start site of SLC15A4 gene

We checked the WES data of the studied family for further variants affecting the same gene of interest. Interestingly, one additional variant in the upstream region of the *SLC15A4* gene showing the same familial segregation as the missense variant (present in the cases and the possible carrier) was identified (12_129308531_C_T; 43 bp upstream of transcription start site, ENST00000266771.5). Functional annotation of the non-coding variant was derived from CADD v1.6 providing a PHRED-like CADD-score of 11.38 (Kircher et al. 2014; Rentzsch et al. 2019). Moreover, the variant was annotated to be located at an active transcription start site according to ChromHmm (TssA, Score = 0.969) and Segway (TSS) (Ernst and Kellis 2012; Hoffman et al. 2012). CADD v1.6 further calculated 52 different overlapping ChIP TFBSs covered by the upstream variant and 115 TFBS peaks when summed over different cell types and tissue.

Using the intersect function of the Bedtools, the non-coding variant was predicted to affect the promoter (129,308,487.129308588) of the *SLC15A4* gene. All described analysis results of the *SLC15A4* upstream variant are summarized in Table 2.

**Table 2:** Analysis results of the *SLC15A4* upstream variant identified in the studied CRC family

Gene name	Chromosomal position	Variant annotation	Pedigree segregation	NFE allele frequency		CADD v1.6						Bedtools intersect		
				ExAC	gnomAD	CADD SCORE	Chromatin state			TFBS	TFBSPeaks^c^	Promoter		
							ChromHMM^a^ state	ChromHMM^a^ score	Segway^b^			Start	End	Strand
SLC15A4	12_129308531_C_T	upstream	III2, III3, III4, III5, IV2	0	3.75 × 10^–3^	11.38	TssA	0.969	TSS	52	115	129,308,487	129,308,588	–

Chromosomal position, variant annotation, pedigree segregation and allele frequency in the Non-Finnish European (*NFE*) population are listed. The PHRED-like CADD score, annotation of the chromatin state and location within transcription factor binding sites (*TFBS*) are derived from CADD v1.6. Affected promoter region according to Bedtools intersect function and SEA, FANTOM5 databases are included with respective start and end positions (Lizio et al. 2015; Wei et al. 2016)

^a^ChromHMM: The ChromHmm score shows the proportion of 127 cell types of the Roadmap Epigenomics project in a particular chromatin state with scores closer to 1 indicating more cell types in the particular chromatin state. The 15 chromatin states are defined as follows: TssA–Active transcription start site (TSS), TssAFInk – Flanking active TSS, TxFlnk–Transcribed at gene 5′ and 3′, Tx–Strong transcription, TxWk–Weak transcription, EnhG–Genic enhancers, Enh–Enhancers, ZNF/Rpts–ZNF genes and repeats, Het–Heterochromatin, TssBiv–Bivalent/Poised TSS/Enhancers, BivFlnk–Flanking bivalent TSS/Enhancer, EnhBiv–Bivalent enhancers, ReprPC–Repressed PolyComb, ReprPCWk–Weak Repressed PolyComb, Quies– Quiescent/low (Ernst and Kellis 2012; Roadmap Epigenomics et al. 2015)

^b^Segway: Segway uses a genomic segmentation method to annotate the chromatin state based on multiple datasets of ChIP-seq experiments. The chromatin states can be annotated as follows: D–dead, F0/1–FAIRE, R0/1/2/4/5–Repressed Region, H3K9me1–histone 3 lysine 9 monomethylation, L0/1–Low zone, GE0/1/2–Gene body (end),TF0/1/2–Transcription factor activity, C0–CTCF, GS–Gene body (start), E/GM–Enhancer/gene middle, GM0/1–Gene body (middle), TSS–Transcription start site, ZnfRpts–zinc finger repeats (Hoffman et al. 2012)

^c^TFBS peaks: The number of overlapping ChIP TFBS peaks summed over different cell types/tissue

In order to identify those transcription factors for which the binding may be affected the most by the variant, we used Jaspar2020 for prediction and comparison of the TFBSs for the wild-type and the mutant sequence of the *SLC15A4* upstream region (Fornes et al. 2020). Whereas most of the identified TFBS were shared by both sequences, nine transcription factors were predicted to bind only to the wild-type sequence, indicating a TFBS disruption by the variant, and eight were predicted to bind only to the mutant sequence, indicating a TFBS creation by the variant (Table 3). One of the identified transcription factors, whose binding site was disrupted was STAT1 which has been established as a favorable prognostic marker in several types of cancers, including CRC (Klampfer 2008; Simpson et al. 2010; Gordziel et al. 2013). Moreover, STAT1 has been proposed as a tumor suppressor particularly in colitis‐associated CRC (Crncec et al. 2018), in turn suggesting a carcinogenic potential of its disruption by the identified upstream variant.

**Table 3:** Summary of transcription factors exclusively targeting either the wild type (*WT*) or the mutant sequence (*MUT*) of *SLC15A4* upstream region

Transcription factor	Targeting	Matrix ID	Relative score^a^	Start	End	Strand	Predicted sequence
MEIS2	WT	MA0774.1	0.84	116	123	+	gggacAGG
NR1D2	WT	MA1532.1	0.81	108	122	+	tgggttctgggacAG
RARA::RXRG	WT	MA1149.1	0.80	109	126	+	gggttctgggacAGGTGA
RBPJ	WT	MA1116.1	0.86	113	122	+	tctgggacAG
RORC	WT	MA1151.1	0.82	110	121	+	ggttctgggacA
SREBF1	WT	MA0595.1	0.80	118	127	–	GTCACCTgtc
STAT1	WT	MA0137.2	0.84	109	123	–	CCTgtcccagaaccc
		MA0137.3	0.88	111	121	+	gttctgggacA
TGIF2LX	WT	MA1571.1	0.81	117	128	–	GGTCACCTgtcc
			0.81	117	128	+	ggacAGGTGACC
TGIF2LY	WT	MA1572.1	0.82	117	128	–	GGTCACCTgtcc
			0.82	117	128	+	ggacAGGTGACC
GRHL2	MUT	MA1105.2	0.83	116	127	+	ggaacAGGTGAC
MYF6	MUT	MA0667.1	0.82	118	127	+	aacAGGTGAC
NFATC2	MUT	MA0152.1	0.90	115	121	–	Tgttcca
PRDM4	MUT	MA1647.1	0.81	114	124	–	ACCTgttccag
SCRT1	MUT	MA0743.1	0.83	114	128	+	ctggaacAGGTGACC
		MA0743.2	0.85	113	128	+	tctggaacAGGTGACC
SCRT2	MUT	MA0744.1	0.85	114	126	+	ctggaacAGGTGA
		MA0744.2	0.85	113	128	+	tctggaacAGGTGACC
TEF	MUT	MA0843.1	0.80	110	121	–	Tgttccagaacc
ZBTB26	MUT	MA1579.1	0.92	107	121	–	Tgttccagaacccag

Respective transcription factor binding sites (*TFBS*) are identified with Jaspar2020 and the default relative profile score threshold of 80%. Matrix ID, relative scores, start and end positions, strand information as well as respective binding sequences are included

^a^A relative score of 1 is representing the maximum likelihood sequence for the motif

## Discussion

Performing WES on a family with CRC aggregation and applying our in-house developed FCVPPv2, we identified two novel heterozygous variants in the *SLC15A4* gene that segregated with the disease in the family. The missense variant, p. Y444C, was predicted to affect the phylogenetically conserved PTR2/POT domain and to have a deleterious effect on the function of the encoded peptide/histidine transporter. The other variant was located in the upstream region of the same gene and it was annotated to affect the promoter region of *SLC15A4* as well as binding sites of several transcription factors. Our findings of two distinct variants in the same gene may indicate a synergistic up-regulation of *SLC15A4* as the underlying genetic cause and implicate this gene for the first time in genetic inheritance of familial CRC.

SLC15A4 belongs to the family of the proton-coupled oligopeptide transporters (POTs) that enable the transfer of histidine and oligopeptides derived from degradation products from inside of the endosome to the cytosol. Since proton dependency implies higher transport activity at low pH levels, endosomal acidification during the maturation to lysosomes is required for substrate uptake by the SLC15A4 transporter (Yamashita et al. 1997; Bhardwaj et al. 2006).

Well-established examples of SLC15A4 substrates are the NOD1 ligands L-Ala-D-Glu-meso-diaminopimelic acid (Tri-DAP) and γ-D-Glu-meso-diaminopimelic acid (iE-DAP), components of the cell wall peptidoglycan of primarily Gram-negative bacteria (Lee et al. 2009; Sasawatari et al. 2011). NOD1 stimulation by DAP induces the activation of nuclear factor-κB and mitogen-activated protein (MAP) kinases and thus the transcription of various genes responsible for innate and adaptive immune responses (Hayden and Ghosh 2004; Franchi et al. 2009). Knockdown of SLC15A4 in HEK293T cells has been shown to lead to decreased nuclear factor-κB activation by the NOD1 ligands (Lee et al. 2009), which was supported by in vivo experiments resulting in loss of Tri-DAP–induced cytokine production in SLC15A4-deficient mice. The same study has further reported an association of SLC15A4 with toll like receptor 9 (TLR9) functions: SLC15A4-deficient dendritic cells showed decreased TLR9-mediated cytokine production which was traced back by the authors to high lysosomal histidine concentrations in the absence of SLC15A4. By being required for TLR9- as well as NOD1-mediated cytokine production, SLC15A4 has been shown to promote Th1-dependent colitis in vivo (Sasawatari et al. 2011).

Since chronic intestinal inflammation has been associated with increased CRC risk, potentially mediated by oxidative DNA damage and innate and adaptive immune responses (Feagins et al. 2009; Ullman and Itzkowitz 2011), SLC15A4 may further play an important role in the initial inflammation-induced colorectal carcinogenesis (https://www.ebi.ac.uk/gwas/efotraits/EFO_0003767; accessed on March 5th, 2021). Based on these findings, we are suggesting a role in CRC susceptibility as well for genetic variation of *SLC15A4*.

Performing WES on a family with CRC aggregation and applying our in-house developed FCVPPv2, we were able to identify a novel heterozygous variant in the coding region of the *SLC15A4* gene. By being present in all four CRC-affected siblings as well as one direct descendant with colorectal polyps, the identified missense variant in *SLC15A4* shows segregation with the disease and a potential for medium-to-high-penetrance susceptibility to CRC in the studied family. Considering the very low allele frequency of the variant in the NFE population of 0 counts in 113,688 alleles, the proposed association of the identified genetic variation with familial CRC is further supported. In silico analyses based on evolutionary conservation, intolerance against functional genetic alterations and deleteriousness led to the prediction of pathogenicity for the missense variant. Snap^2^ further predicted an effect on protein function by the missense variant leading to the amino acid substitution Y444C in *SLC15A4*. Considering all analyses, we propose an up-regulating mode of action for the identified missense variant on SLC15A4 protein level.

Interestingly, we identified another variant with the same familial segregation in the upstream region of the *SLC15A4* gene (12_129308531_C_T; 43 bp upstream of transcription start site, ENST00000266771.5). GnomAD browser reported an allele frequency of 3.754 × 10^–3^ in the NFE population. Taking this relatively high frequency into account, high penetrance and thus strong functional consequence of the upstream variant by itself may not be expected. Nevertheless, synergistic effects of both variants occurring in the same gene have to be considered: The upstream variant may have an enhancing impact on SLC15A4 protein expression, potentially of minor relevance when solely occurring but which may reinforce the postulated up-regulating mode of action of the *SLC15A4* coding variant in the course of colorectal carcinogenesis. In order to confirm the proposed mode of function, we assessed the upstream variant for potentially influencing gene transcription. According to our analysis, the upstream variant was annotated to be located at an active transcription start site affecting the promoter region of the *SLC15A4* gene. In particular, binding sites of 17 different transcription factors were predicted to be exclusive for either the wild type or the mutant sequence due to the identified upstream variant, representing a potential mechanism of enhancing gene transcription. Whether the variant potentially destroys TFBSs for transcriptional repressors or creates new TFBSs for transcriptional activators, remains unclear and requires further functional experiments. By providing a list of TFBSs and potential transcriptional repressors or activators, including the tumor suppressor STAT1, we aim to lay the foundation for functional validation of the regulatory impact of the upstream variant and instigate further research in this field. Thus, we hope to facilitate a better understanding of the identified upstream variant in the context of *SLC15A4* gene regulation in particular and of the postulated synergistic model of coding and non-coding variants in cancer predisposition in general.

Certainly, the confined number of analyzed family members and particularly healthy controls has to be taken into account as a statistical limitation of this study when finally interpreting the described results. Due to lack of availability of additional blood samples, the inclusion of further family members in our analyses was not feasible to increase the statistical power. We met this limitation to some extent by considering the allele frequencies of the identified variants in large populations according to gnomAD (Karczewski et al. 2019) and TOPMed data (Li et al. 2020; Taliun et al. 2021). Further validation of the identified variants has been provided by the large-scale WES data of UK Biobank, reporting statistically significant gene-phenotype associations of the *SLC15A4* gene and the clinical phenotypes of malignant neoplasms in the colon and rectum (Wang et al. 2021).

By identifying germline variants in the *SLC15A4* gene in familial CRC, we implicated this gene for the first time in genetic inheritance of a malignancy, expanding its role from a potential CRC marker in quantitative fecal tests to a potential marker of CRC susceptibility in genetic testing. However, the results of this study need to be further replicated in validation cohorts and validated using experimental approaches in cell lines.

## Supplementary Information

Below is the link to the electronic supplementary material.

Supplementary file1 (DOCX 259 KB)

## Data Availability

Unfortunately, for reasons of ethics and patient confidentiality, we are not able to provide the sequencing data into a public database. The data underlying the results presented in the study are available from the corresponding author or from Dr. Asta Försti (Email: a.foersti@kitz-heidelberg.de).
