# Supplementary material for: Whole exome sequencing identifies novel germline variants of SLC15A4 gene as potentially cancer predisposing in familial colorectal cancer
Source: Mol Genet Genomics. 2022 May 13;297(4):965–79. doi: 10.1007/s00438-022-01896-0 (PMC9250485; doi:10.1007/s00438-022-01896-0)
Supplement: Supplementary file 1 — Supplementary file1 (DOCX 259 KB) [file 438_2022_1896_MOESM1_ESM.docx]

**Online Resources**

**Article title:** Whole exome sequencing identifies novel germline variants of *SLC15A4* gene as potentially cancer predisposing in familial colorectal cancer

**Journal:** Molecular Genetics and Genomics

**Authors:** Diamanto Skopelitou ^1,2^, Aayushi Srivastava ^1,2^, Beiping Miao ^1^, Abhishek Kumar ^1,3,4^, Dagmara Dymerska ^5^, Nagarajan Paramasivam ^6^, Matthias Schlesner ^7^, Jan Lubinski ^5^, Kari Hemminki ^1,8,^ Asta Försti ^1^ and Obul Reddy Bandapalli ^1,2,^*

^1^ Molecular Genetic Epidemiology, German Cancer Research Center (DKFZ), Heidelberg, Germany

^2^ Medical Faculty Heidelberg, Heidelberg University, Heidelberg, Germany

^3^ Institute of Bioinformatics, International Technology Park, Bangalore, India

^4^ Manipal Academy of Higher Education (MAHE), Manipal 576104, Karnataka, India

^5^ Department of Genetics and Pathology, Pomeranian Medical University in Szczecin, Poland

^6^ Computational Oncology, Molecular Diagnostics Program, National Center for Tumor Diseases (NCT), Germany

^7^ Bioinformatics and Omics Data Analytics, German Cancer Research Center (DKFZ), Heidelberg, Germany

^8^ Faculty of Medicine and Biomedical Center in Pilsen, Charles University in Prague, 30605 Pilsen, Czech Republic

* Correspondence: o.bandapalli@kitz-heidelberg.de; Tel.: +49-6221-421809

**1** Details of multiple sequence alignment. Selected representative species with their respective NCBI (National Center for Biotechnology Information) accession numbers for *SLC15A4* and *PTGES* gene are summarized.

| Species | Common Name | NCBI Accession Numbers | |
| --- | --- | --- | --- |
|  |  | ***SLC15A4*** | ***PTGES*** |
| Homo sapiens | **Human** | NP_663623.1 | NP_004869.1 |
| Mus musculus | **Mouse** | NP_598656.1 | NP_071860.1 |
| Rattus norvegicus | **Norway rat** | NP_653359.1 | NP_067594.1 |
| Oryctolagus cuniculus | **Rabbit** | XP_002722114.3 | XP_017194907.1 |
| Gallus gallus | **Chicken** | XP_415099.3 | NP_001181912.1 |
| Pan troglodytes | **Chimpanzee** | XP_016780109.2 | XP_009455764.2 |
| Pongo abelii | **Sumatran orangutan** | XP_002824029.2 | XP_002820341.1 |
| Callithrix jacchus | **Marmoset** | XP_009003149.3 | XP_002743420.1 |
| Macaca mulatta | **Rhesus monkey** | XP_014965182.2 | NP_001248097.1 |
| Tupaia chinensis | **Chinese tree shrew** | XP_006147431.2 | XP_006154174.2 |
| Sapajus apella | **Tufted capuchin** | XP_032100059.1 | XP_032113453.1 |
| Bos taurus | **Cattle** | NP_001095397.2 | NP_776868.1 |

**
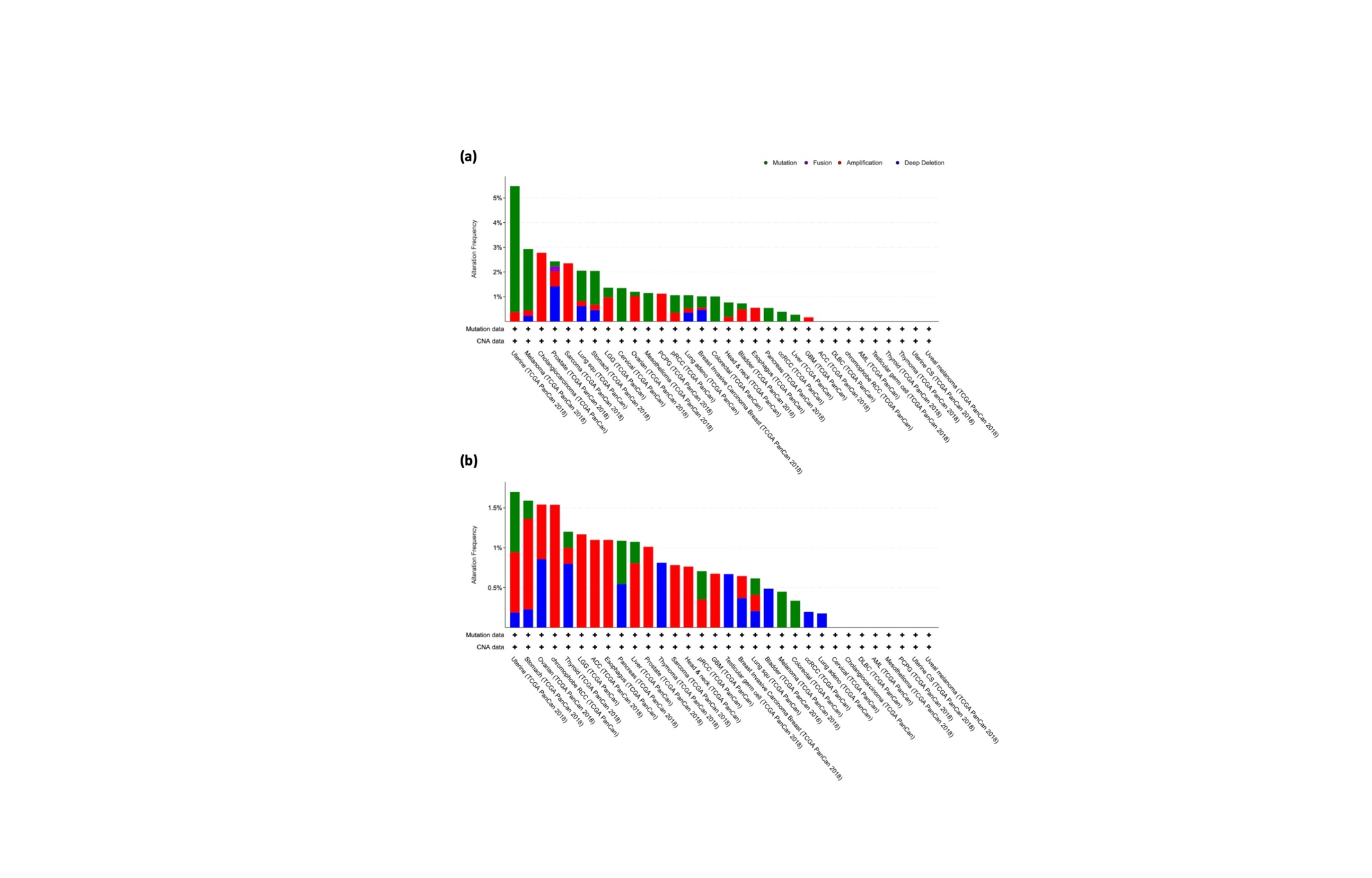
2** Histograms of alteration frequencies in different cancers extracted from cBioPortal (www.cbioportal.org) on 13th of December 2020 according to the TCGA PanCancer data **(a)** *SLC15A4* **(b)** *PTGES*
